# Prognostic Nutritional Index as a Novel Predictor of In-Stent Restenosis: A Retrospective Study

**DOI:** 10.3390/medicina59040663

**Published:** 2023-03-27

**Authors:** Ahmet Balun, Alkame Akgümüş, Kerem Özbek, Zehra Güven Çetin

**Affiliations:** 1 Department of Cardiology, Bandırma Onyedi Eylül University, 10200 Balıkesir, Turkey; aakgumus@bandirma.edu.tr; 2 Department of Cardiology, Ankara City Hospital, 06800 Ankara, Turkey; keremozbek@dr.com (K.Ö.); mdzehragc@gmail.com (Z.G.Ç.)

**Keywords:** in-stent restenosis, malnutrition, nutritional status, prognostic nutritional index

## Abstract

*Background and Objectives*: In-stent restenosis (ISR) is a major problem in patients undergoing percutaneous coronary intervention. The prognostic nutritional index (PNI) is a nutritional status score used in the literature to determine the prognosis of coronary artery disease. In this study, we aimed to investigate the effect of preprocedural PNI values on the risk of ISR in patients with stable coronary artery disease who underwent successful percutaneous coronary intervention. *Materials and Methods*: This retrospective study included 809 patients. Stent restenosis was evaluated in the follow-up coronary angiography of the patients due to stable angina pectoris or acute coronary syndrome. The patients were divided into two groups based on those with (*n* = 236) and without (*n* = 573) in-stent restenosis, and their nutritional status was compared with PNI. The PNI values before the first angiography of the patients were calculated. *Results*: The mean PNI score was significantly lower in patients with ISR than in those without ISR (49.5 vs. 52.3, *p* < 0.001). Concerning the results of the Cox regression hazard model for predictors of ISR, PNI was significantly associated with the development of ISR (HR = 0.932, 95% CI: 0.909–0.956, *p* < 0.001). In addition, stent type, stent length, and diabetes mellitus were associated with the development of ISR. *Conclusions*: A low PNI value indicates poor nutritional status, which is thought to accelerate inflammation processes and cause atherosclerosis and ISR.

## 1. Introduction

Coronary artery disease is the leading cause of death worldwide, and its incidence is increasing. Percutaneous coronary intervention has become the essential treatment modality for coronary artery disease. Stenosis of stents implanted with percutaneous coronary intervention is an important problem. There is a complex and incompletely explained mechanism of stent restenosis. Understanding the mechanism of restenosis and predicting restenosis may contribute to interventional and medical treatment modalities of patients. Although in-stent restenosis (ISR) in old-generation bare metal stents has been reduced with new-generation drug-eluting stents, ISR continues to be an important problem [1]. With the development of stent technology, new antiplatelet strategies are being employed to prevent ISR. Although we have this technology available to prevent ISR, it has become important to define the risks that will cause ISR and to take precautions according to these risks.

Coronary artery disease is a progressive disease, and atherosclerosis progresses in the stent as well as in segments without revascularization. Although ISR is a result of endothelial dysfunction and endothelial rupture due to vascular damage in the early period, it is caused by neointimal hyperplasia and atherosclerosis in the late period [2]. Clinical, biological and genetic risk factors for stent restenosis have been identified in previous studies. The most clinically significant risk factor is diabetes mellitus. The development of atherosclerosis is accelerated due to endothelial dysfunction caused by hyperglycemia and coronary artery disease. Considering the biological risk factors, the most important one is inflammation. The inflammatory process is involved in the development of neointimal hyperplasia and atherosclerosis, which causes stent restenosis [3]. With the increase in the use of drug-eluting stents, stent restenosis has also decreased. Antiproliferative agents are used in drug-eluting stents, and they have an antimitotic effect on them. Because of this antimitotic effect, less incidence of stent restenosis has proven that the inflammatory process is effective in stent restenosis.

Nutritional status is an important condition that affects inflammation [4]. Body mass index gives information about the nutritional condition in the clinic. Dietary components can directly affect inflammation. For this reason, there is a need for additional parameters that will affect the nutritional status clinically, apart from the body mass index calculated by body weight and height. It has been shown in previous studies that nutritional status may adversely impact prognosis by affecting inflammatory processes in heart failure and coronary artery disease [5,6]. Major adverse cardiac events are more common in the follow-up of patients with poor nutritional status and therefore have a worse prognosis. Many tools have been developed to measure nutritional status, among which the prognostic nutritional index (PNI) and controlling nutritional status (CONUT) score are associated with determining a prognosis in stable coronary artery disease and acute coronary syndrome [6,7]. Poor nutritional status is thought to accelerate atherosclerosis by increasing inflammation. Although PNI has been shown to affect prognosis in coronary artery disease, there are no studies on stent restenosis that can be caused by inflammation influenced by nutritional status. The aim of this study is to investigate the effect of pre-procedural PNI values on the risk of developing ISR in patients who underwent successful percutaneous coronary intervention due to stable coronary artery disease.

## 2. Materials and Methods

A total of 809 patients were included in this single-center retrospective study. The study was conducted according to the principles outlined in the Declaration of Helsinki and was approved by the local ethics committee. The patients included in the study consisted of patients who underwent percutaneous coronary intervention due to stable angina pectoris and successful stent implantation between October 2015 and May 2022. Since albumin is an acute phase reactant and lymphocyte count may be affected in acute coronary syndromes, patients with stable coronary artery disease were included in the study. Patients whose stent patency was evaluated after repeated coronary angiography due to stable angina pectoris, unstable angina pectoris, or acute myocardial infarction were included in the study. The patients were divided into two groups based on those with and without in-stent restenosis, and their nutritional status was compared with PNI and CONUT. All patients received statins and 12 months of dual antiplatelet therapy. The detailed exclusion criteria were as follows: patients under 18 years of age, combined use of anticoagulants, heart failure, history of previous coronary artery bypass surgery, a history of severe intolerance or allergy to one of the drugs (acetylsalicylic acid, clopidogrel, ticagrelor), active infection at the time of first angiography, a history of hematologic disease, chronic kidney disease, severe liver function disorder, malignancy, and chronic inflammatory disease. 

The clinical, demographic, and laboratory results were obtained from the hospital’s electronic record system. CONUT score and PNI were calculated retrospectively according to laboratory data, and venous blood samples of the patients were taken before the first angiography. Successful stent implantation was evaluated as providing Thrombolysis in Myocardial Infarction grade-3 flow without major or minor complications and reducing the stenosis diameter to less than 20% [8]. ISR was defined as stenosis of more than 50% involving the proximal and distal 5 mm segments of the stent [9]. Evaluation of ISR was performed using a quantitative angiography method by two experienced interventional cardiologists. The drug-eluting stents used in the study are new-generation drug-eluting stents. In order to prevent cardiovascular diseases, glycemic control of patients with diabetes mellitus is important. In our study, for patients diagnosed with diabetes mellitus, a HbA1c level < 7% was defined as controlled diabetes mellitus (97).

The PNI was calculated using the formula (10 × albumin (gr/dL)) + (0.005 × total lymphocyte count (per mm^3^)) from the laboratory values of the patients before their first coronary angiography. A low PNI is suggestive of malnutrition. The CONUT score was calculated in the same way as in previous studies using albumin levels, total cholesterol levels, and total lymphocyte counts [10]. If the patient’s serum albumin is ≥3.5 g/dL, total cholesterol ≥ 180 mg/dL, and total lymphocytes ≥ 1600 count/mL, then the CONUT score is 0, which indicates good nutritional status. A decrease in serum albumin level, lymphocyte count and total cholesterol level increases the CONUT score, and a high CONUT score indicates poor nutritional status. Based on previous studies on nutritional status, the patients were divided into groups according to their CONUT scores; those with a CONUT score of 0–1 were considered normal, those with scores of 2–4 were considered to have mild malnutrition, and those with scores ≥ 5 were considered to have moderate–severe malnutrition [11].

### Statistical Analysis

For descriptive statistics, the mean ± standard deviation was used to provide continuous data with a normal distribution. A median with interquartile range values was applied for continuous variables without normal distribution. Numbers and percentages were used for categorical variables. Student’s *t*-test analyzed the continuous data with a normal distribution. The Mann–Whitney U test compared two independent groups in which numerical variables had no normal distribution. The Kruskal–Wallis test compared more than two independent groups in which numerical variables were without normal distribution. The Pearson chi-square and Fisher’s exact tests were used to compare the differences between categorical variables in 2 × 2 tables. We used cox regression analysis with the entry model to show the independent predictors of ISR. The clinically essential factors in the univariate regression model with a *p*-value of <0.1 were included in the multivariate analysis. SPSS software (version 17.0, SPSS Inc., Chicago, IL, USA) was used for the statistical analyses. The significance level (*p*-value) was determined to be 0.05 in all statistical analyses.

## 3. Results

There were 809 patients in the study group. The number of patients with and without ISR was 236 (29.2%) and 572 (70.8%). The patient groups were similar in terms of demographic variables (Table 1). We detected no significant differences in the clinical variables except for the incidence of diabetes mellitus in the patient groups. Patients with ISR were more frequently diabetic than those without ISR (36.4% vs. 25.8%, *p* = 0.002). The other clinical variables, including smoking and hypertension, were similar between the patient groups. The glycemic controls of patients with diabetes mellitus were examined. Uncontrolled hyperglycemia was higher in the group with ISR, but there was no statistically significant difference (44.2% vs. 33.1%, *p* = 0.061). The body mass indexes (BMI) of the patients were compared, and no significant difference was found between the two groups (23.2 ± 2.7 vs. 23.0 ± 2.6, *p* = 0.422).

Moreover, Table 1 presents the angiographic characteristics of the patient groups. A bare metal stent was used in most of the patients in both groups. The technical features of the stents, except for the diameter, were similar between the patient groups. The diameter of the stents was significantly larger in patients without ISR than in those with ISR (3.27 ± 0.50 vs. 3.14 ± 0.48 mm, *p* < 0.001). The stent length was longer with ISR, but there was no statistically significant difference (18.1 ± 5.8 vs. 19.0 ± 6.1 mm). The left anterior descending artery was the most common location for stent application, and it was used in 50.8% of the patients without ISR and 44.5% with ISR. Comparisons of the localizations of the stents were similar between the patient groups. There was no significant difference between the groups regarding the period between the two angiograms.

A comparison of the laboratory measurements in the patient groups is given in Table 2. There were significant differences in the values of lymphocyte count, albumin, and high-density lipoprotein (HDL) between the patients with and without ISR (*p* = 0.026, *p* < 0.001, and *p* < 0.001). The mean serum albumin level was significantly lower in patients with ISR than in those without ISR (3.9 ± 0.3 mg/dL vs. 4.1 ± 0.3 mg/dL). The mean lymphocyte count was significantly higher in patients without ISR than in those with ISR (2179 ± 872 vs. 2036 ± 730 µ/µL). There was no significant difference between the mean LDL levels of the patients, but the mean HDL was found to be higher in the ISR (−) group. There was no significant difference in mean HbA1c levels between the two groups. 

The other laboratory variables were similar in the patient groups. The mean PNI score was significantly lower in patients with ISR than in those without ISR (49.5 ± 5.1 vs. 52.3 ± 5.8, *p* < 0.001). The median CONUT score and the grouping based on its value revealed no significant differences between the patient groups. In the nutritional status groups created according to the CONUT score, more than half of the ISR + and ISR- were in the normal nutrition group.

The results of the Cox regression hazard model for predictors of ISR are shown in Table 3. In the Cox regression models, drug-eluting stent (HR = 0.622, 95% CI: 0.452–0.855, *p* = 0.003), stent length (HR = 1.025, 95% CI: 1.003–1.048, *p* = 0.028), diabetes mellitus (HR = 1.408, 95% CI: 1.068–1.858, *p* = 0.015), HDL (HR = 0.953, 95% CI: 0.941–0.966, *p* < 0.001) and PNI (HR = 0.932, 95% CI: 0.909–0.956, *p* < 0.001) were significantly associated with the development of ISR.

## 4. Discussion

To our knowledge, this study is the first in the literature to examine the relationship between nutritional status and ISR. In our study, nutritional status was evaluated with PNI, and it was shown that the nutritional status of those with a high PNI value was better and significantly reduced ISR.

Since percutaneous coronary intervention is the key treatment method for coronary artery disease, the number of patients with stent implantation is increasing; therefore, ISR, which is an important problem in these patients, is also increasing. Previous studies have shown that atherosclerosis and intimal hyperplasia, and therefore inflammation, play a role in the pathophysiology of ISR [2]. 

Malnutrition is associated with poor outcomes in chronic diseases, suggesting that malnourished patients are vulnerable [12]. This vulnerability caused by malnutrition may be due to the inflammatory potential of the diet. Malnutrition is also important as it is a modifiable risk factor. Previous studies have shown that malnutrition increases inflammation [13]. Recently developed nutritional status scores have been used in many studies showing malnutrition and inflammatory processes and have been associated with poor prognosis in patients with cancer, chronic kidney disease, coronary artery disease, heart failure, ischemic cerebrovascular event, and coronary collateral development [6,14,15,16,17]. Nutritional status in these studies prolonged hospital stays and was associated with postoperative complications. PNI has been shown to be a predictor of cardiovascular and all-cause mortality. This has been attributed to the increase in inflammation in malnutrition.

In our study, we used PNI and CONUT scores, which indicate nutritional status. While albumin and lymphocyte counts are used to calculate PNI, albumin, lymphocyte count, and total cholesterol level are used to calculate the CONUT score. Albumin is both a protein that indicates nutritional status and an acute-phase reactant that indicates inflammation. Previous studies have shown that albumin is also effective for platelet aggregation [18]. Albumin is an important protein for atherosclerosis and coronary artery disease due to its anti-inflammatory effect, as well as its inhibiting platelet aggregation and anticoagulant effects. Excess albumin decreases platelet aggregation in plasma, and albumin deficiency increases platelet aggregation, which may be associated with poor prognosis in sepsis and chronic diseases. Decreased serum albumin levels may trigger inflammation that causes coronary artery disease, as it activates proinflammatory cytokines. It has been previously shown that low serum albumin is a risk factor for cardiovascular diseases, and this risk is independent of inflammation and body mass index [19]. Lymphocytes are a marker of inflammation and regulate the inflammatory response in the atherosclerotic process [20]. The development of atherosclerosis is a long inflammatory process. In this process, dysfunction of endothelial cells leads to the adhesion of leukocytes, which increases lipid accumulation in the intima and initiates atherosclerosis. The PNI and CONUT scores, which evaluate nutritional status within these parameters, are valuable in terms of inflammation and malnutrition.

As the PNI value, which indicates nutritional status, decreases, it indicates poor nutritional status. In our study, ISR was observed more frequently in those with low PNI values. In addition, the reason that patients with poor nutritional status had increased ISR may be due to the mechanism of inflammation. Wada et al. reported the relationship between PNI value and long-term clinical outcomes in patients with stable angina pectoris and percutaneous coronary intervention [6]. In this study, the incidence of major adverse cardiac events may have increased because patients with poor nutritional status had worse characteristics in terms of age and comorbid diseases. However, information about ISR was not provided in this study. The relationship between PNI and ISR, which we demonstrated in our study, may be related to these long-term clinical outcomes. 

The CONUT score is associated with poor clinical outcomes in coronary artery disease, such as PNI [21]. There are not enough studies on the effect of CONUT score on atherosclerosis and stent restenosis. It can be thought that those with a normal nutritional status according to the CONUT score may have higher cholesterol levels, which may lead to higher statin treatment in these patients. This suggests that less stent restenosis may be observed in these patients. In our study, no significant difference was found between those with and without stent restenosis in terms of CONUT value. The reason for this is considered to be that the total cholesterol level is included in the calculation of the CONUT score, unlike the PNI. Because a high cholesterol level can accelerate atherosclerosis, it increases ISR [22]. In Gordon et al.’s study, HDL was shown to be protective in terms of atherosclerosis; similarly, less ISR was observed in patients with high HDL in our study [23]. HDL prevents the development of atherosclerosis with its reverse cholesterol transport and antioxidant and anti-inflammatory properties.

BMI is mainly used to evaluate nutritional status, while PNI also highlights the immunonutritional status. Although obesity has a protective effect in cardiovascular diseases, it has been reported that recurrent revascularizations are increased in patients with recurrent severe obesity [24]. In our study, while there was no significant difference between the mean body mass indexes of the groups, a significant difference was found between the prognostic nutritional indexes. This shows that the immunonutritional status comes to the fore in stent restenosis.

In their study, Ullrich et al. showed that the presence of diabetes mellitus increased ISR [25]. The most clinically important risk factor for stent restenosis is diabetes mellitus. DM causes microvascular and cardiovascular complications. The proinflammatory state caused by hyperglycemia in DM may increase stent restenosis by causing endothelial dysfunction. Long-term glycemic control is necessary to prevent cardiovascular complications. HbA1c < 7% was targeted for this glycemic control. Although it has been reported that high HbA1c is a risk for ISR in previous studies, no significant difference was found between the groups in our study [26]. This showed that uncontrolled diabetes is prevented in diabetic patients due to more regular controls after percutaneous coronary intervention. In our study, ISR was observed more frequently in patients with diabetes. 

In addition to clinical and inflammatory causes of stent restenosis, there are also procedural determinants of percutaneous coronary intervention. The coronary target lesion is in the bifurcation region, the vessel diameter is small, and the lesion is long. Bare metal stents and long stents are more susceptible to restenosis. The fact that bare metals are prone to this restenosis has increased the use of drug-eluting stents. Less restenosis is observed in the new-generation drug-eluting stents compared to the old generation. In our study, it is predicted that the use of drug-eluting stents may reduce the risk of stent restenosis. Due to the use of new-generation DES in the center where the study was conducted, no comparison could be made between the old-generation DES and the new-generation DES in terms of stent restenosis. Zbinden et al. showed that ISR increases as the stent diameter decreases and its length increases and that there is less restenosis in drug-eluting stents [27]. Similarly, in our study, it was observed that the stent diameter was significantly narrower in the group with ISR. It was also observed that stent length is an independent risk factor for ISR. The absence of a difference between drug-eluting stents and bare metal stents may be due to less use of drug-eluting stents in these patients.

This study has several limitations, of which the main one was its retrospective and single-center design. Moreover, the limited prediction of nutritional status by the PNI was also a limitation. Nutritional indexes may differ in different ethnic populations. Since the prognostic nutritional index is calculated with the initial reference values, the change in the nutritional values of the patients is unknown. Although there are many predictors of stent restenosis, this study is for hypothesis generation, and experimental studies are needed. Prospective studies are needed to show the effects of nutritional status correction on stent restenosis.

## 5. Conclusions

In conclusion, this study showed that PNI, which indicates nutritional status, can predict ISR. A low PNI value indicates poor nutritional status, which is thought to accelerate inflammation processes and cause atherosclerosis and ISR.

## Figures and Tables

**Table 1:** Demographic, clinical and angiographic characteristics of the patients with and without restenosis.

	ISR − (*n* = 573)	ISR + (*n* = 236)	*p*-Value
Age	61.3 ± 11.1	60.2 ± 10.4	0.195
Sex, Male *n* (%)	408 (71.2)	164 (69.5)	0.343
Smoking, *n* (%)	137 (23.9)	68 (28.8)	0.086
Diabetes mellitus, *n* (%)	148 (25.8)	86 (36.4)	0.002
Uncontrolled hyperglycemia, *n* (%)	49 (33.1)	38 (44.2)	0.061
Hypertension, *n* (%)	237 (41.4)	96 (40.7)	0.461
Body mass index (kg/m^2^)	23.2 ± 2.7	23.0 ± 2.6	0.422
**Types of stent**			
Bare metal	373 (65.1)	167 (70.8)	0.070
Drug-eluting	200 (34.9)	69 (29.2)	
**Technical features of stents**			
Diameter (mm)	3.27 ± 0.50	3.14 ± 0.48	0.001
Length (mm)	18.1 ± 5.8	19.0 ± 6.1	0.071
Number of stent	1.0 (1.0–1.0)	1.0 (1.0–1.0)	0.572
**Target coronary artery**			
LMCA	0 (0.0)	1 (0.4)	0.096
LAD artery	291 (50.8)	105 (44.5)	
CX artery	108 (18.8)	42 (17.8)	
RCA	174 (30.4)	88 (37.3)	
Period between 2 coronary angiographies, days	550 (502–690)	535 (501–678)	0.757

ISR: In-stent restenosis, LMCA: left main coronary artery, LAD: left anterior descending artery, CX: circumflex artery, RCA: right coronary artery.

**Table 2:** Comparison of the laboratory measurements in the patient groups.

	ISR − (*n* = 573)	ISR + (*n* = 236)	*p*-Value
White blood cell count (µ/µL)	10,398 ± 3898	10,652 ± 3480	0.385
Lymphocyte count (µ/µL)	2179 ± 872	2036 ± 730	0.026
Neutrophil count (µ/µL)	7178 ± 3677	7486 ± 3201	0.262
Hemoglobin (g/dL)	14 ± 1.7	13.8 ± 1.8	0.089
Platelet count (×10^3^/µL)	231.7 ± 80.6	243.7 ± 79.1	0.053
Creatinine (mg/dL)	0.92 (0.81–1.08)	0.91 (0.79–1.14)	0.131
Uric acid (mg/dL)	5.7 ± 1.1	5.7 ± 1.1	0.741
Albumin (g/dL)	4.1 ± 0.3	3.9 ± 0.3	<0.001
HbA1c (%)	6.6 ± 1.2	6.7 ± 1.4	0.063
Total cholesterol (mg/dL)	167.5 ± 33.9	174.3 ± 38.3	0.013
High-density lipoprotein (mg/dL)	42.9 ± 10.9	34.5 ± 10.9	<0.001
Low-density lipoprotein (mg/dL)	110.9 ± 34.4	108.9 ± 36.8	0.548
PNI	52.3 ± 5.8	49.5 ± 5.1	<0.001
CONUT score	1.0 (0.0–2.0)	1.0 (0.0–2.0)	0.759
Normal	357 (62.3)	134 (56.8)	0.129
Mild	207 (36.1)	94 (39.8)	
Moderate–severe	9 (1.6)	8 (3.4)	

PNI: prognostic nutrition index, CONUT: controlling nutritional status.

**Table 3:** Cox proportional hazard model for predicting the development of restenosis.

	Univariate	Multivariate
Dependent: Restenosis	HR (95% CI, *p*-Value)	HR (95% CI, *p*-Value)
Smoking	1.268 (0.956–1.681, *p* = 0.110)	
Hemoglobin	0.901 (0.838–0.968, *p* = 0.004)	0.971 (0.898–1.050, *p* = 0.466)
Creatinine	1.586 (0.911–2.760, *p* = 0.103)	
HbA1c	1.043 (0.947–1.149, *p* = 0.389)	
Drug-eluting stent	0.724 (0.543–0.964, *p* = 0.027)	0.622 (0.452–0.855, *p* = 0.003)
Stent length	1.022 (1.000–1.043, *p* = 0.045)	1.025 (1.003–1.048, *p* = 0.028)
Stent diameter	0.710 (0.534–0.944, *p* = 0.019)	0.744 (0.545–1.016, *p* = 0.063)
Diabetes mellitus	1.487 (1.138–1.942, *p* = 0.004)	1.408 (1.068–1.858, *p* = 0.015)
PNI	0.863 (0.830–0.896, *p* < 0.001)	0.932 (0.909–0.956, *p* < 0.001)
Low-density lipoprotein	1.002 (0.997–1.007, *p* = 0.401)	
High-density lipoprotein	0.947 (0.934–0.960, *p* < 0.001)	0.953 (0.941–0.966, *p* < 0.001)

HR: hazard ratio, CI: confidence interval.

## Data Availability

Data are available upon request from the corresponding author due to privacy issues.
